# Microbiomes of Caribbean Octocorals Vary Over Time but Are Resistant to Environmental Change

**DOI:** 10.3389/fmicb.2020.01272

**Published:** 2020-06-12

**Authors:** Mark McCauley, Colin R. Jackson, Tamar L. Goulet

**Affiliations:** Department of Biology, The University of Mississippi, University, MS, United States

**Keywords:** coral, gorgonian, microbiome, bacteria, temperature, UVR, nitrogen, phosphorus

## Abstract

The bacterial microbiome is an essential component of many corals, although knowledge of the microbiomes in scleractinian corals far exceeds that for octocorals. This study characterized the bacterial communities present in shallow water Caribbean gorgonian octocorals over time and space, in addition to determining the bacterial assemblages in gorgonians exposed to environmental perturbations. We found that seven shallow water Caribbean gorgonian species maintained distinct microbiomes and predominantly harbored two bacterial genera, *Mycoplasma* and *Endozoicomonas.* Representatives of these taxa accounted for over 70% of the sequences recovered, made up the three most common operational taxonomic units (OTUs), and were present in most of the gorgonian species. Gorgonian species sampled in different seasons and/or in different years, exhibited significant shifts in the abundances of these bacterial OTUs, though there were few changes to overall bacterial diversity, or to the specific OTUs present. These shifts had minimal impact on the relative abundance of inferred functional proteins within the gorgonian corals. Sequences identified as *Escherichia* were ubiquitous in gorgonian colonies sampled from a lagoon but not in colonies sampled from a back reef. Exposure to increased temperature and/or ultraviolet radiation (UVR) or nutrient enrichment led to few significant changes in the gorgonian coral microbiomes. While there were some shifts in the abundance of the prevalent bacteria, more commonly observed was “microbial switching” between different OTUs identified within the same bacterial genus. The relative stability of gorgonian coral bacterial microbiome may potentially explain some of the resistance and resilience of Caribbean gorgonian corals against changing environmental conditions.

## Introduction

Scleractinian (hard) corals and octocorals (soft corals, sea pens, gorgonians) contain organisms such as photosynthetic dinoflagellates [family Symbiodiniaceae; LaJeunesse et al. (2018)], corallicolids (Kwong et al., 2019), bacteria (Rohwer et al., 2002), Archaea (Kellogg, 2004), and fungi (Kendrick et al., 1982) that together constitute the holobiont. For over 100 years, the symbiosis between corals and the endosymbiotic Symbiodiniaceae commanded center stage. The bacterial consortium in the microbiome, however, also plays a significant role in the health, functioning and survival of coral colonies (Ainsworth et al., 2010; Ainsworth and Gates, 2016; Bourne et al., 2016). For example, bacteria in the surface mucus protect the coral holobiont through the production of antimicrobial compounds and by competing with external opportunistic microbes for available niches (Rypien et al., 2010; Krediet et al., 2013; Frydenborg et al., 2014). Furthermore, metagenomic sequencing revealed that bacterial communities participate in the fixation and breakdown of carbon and nitrogen (Wegley et al., 2007; Kimes et al., 2010; Yang et al., 2013) and in the cycling of sulfur and phosphorus (Gilbert et al., 2009; Raina et al., 2010).

The bacterial communities within corals are significantly different in composition and density from those in the surrounding water column (Frias-Lopez et al., 2002; Garren and Azam, 2010). Specificity exists, whereby the microbiomes of colonies of a given coral species share many bacterial species (Roder et al., 2015; Wessels et al., 2017). These bacteria are commonly present across reefs at the same depth (Lee et al., 2012; Ainsworth et al., 2015), but can vary between depths (Glasl et al., 2017a), and seasonally (Kimes et al., 2013; Roder et al., 2015). Corals also have transitory bacterial assemblages that can differ within (La Rivière et al., 2013; Sweet et al., 2017) and between (Lee et al., 2012) reefs, vary between geographical locations, and are associated with specific local conditions (McDevitt-Irwin et al., 2017). The diversity and composition of these bacterial communities may be influenced by coral holobiont parameters such as protein content and the abundance of Symbiodiniaceae (Bourne et al., 2016; Grottoli et al., 2018). Because specific and transient microbial bacteria vary, van de Water et al. (2017, 2018a) suggested that the coral holobiont should be viewed as a species continuum rather than an absolute.

Changes in environmental conditions can alter the microbiome, potentially compromising the holobiont (reviewed in Ainsworth and Gates, 2016; Glasl et al., 2017b; Morrow et al., 2018). Elevated seawater temperatures, nutrient enrichment and ocean acidification can all affect coral bacterial communities (D’Angelo and Wiedenmann, 2014; Stocker, 2014; Häder et al., 2015; Hoegh-Guldberg et al., 2017; Wessels et al., 2017). For example, exposure to elevated temperatures can induce a shift from beneficial bacterial communities to opportunistic populations (Bourne et al., 2008; Garren et al., 2009; Littman et al., 2010; Kelly et al., 2014) which include a greater abundance of commensal or parasitic heterotrophic bacterial species (Thurber et al., 2009; Littman et al., 2011). These changes to the coral bacterial community can lead to increased susceptibility of the coral to diseases and infections (Thurber et al., 2009; Vezzulli et al., 2010; Shirur et al., 2016), lesions and tissue loss (Cárdenas et al., 2011; Meyer et al., 2014) and to bleaching (loss of Symbiodiniaceae) or even colony mortality (Ziegler et al., 2017; Morrow et al., 2018). If not fatal, these bacterial community shifts may be transitory, with the pre-stress bacterial community subsequently re-establishing itself once the stressful conditions have been alleviated (Pootakham et al., 2017).

Despite bacterial microbiome presence in both scleractinian and octocoral holobionts, knowledge about the relationships between octocorals and their bacterial microbiome is sparse. With next generation sequencing, the bacterial microbiomes of eight temperate and deep sea gorgonian species, all of whom do not host Symbiodiniaceae, were characterized (Vezzulli et al., 2013; van de Water et al., 2017, 2018b; Goldsmith et al., 2018; Quintanilla et al., 2018). In addition, the microbiomes of seven gorgonian species that host endosymbiotic Symbiodiniaceae, including five species from shallow water Caribbean reefs, were described (e.g., Sunagawa et al., 2010; Tracy et al., 2015; McCauley et al., 2016; Shirur et al., 2016). Under ambient conditions, bacterial microbiomes of gorgonian corals that host Symbiodiniaceae are dominated by members of the phyla Proteobacteria, Tenericutes, and Bacteroidetes (Tracy et al., 2015; Holm and Heidelberg, 2016; McCauley et al., 2016; Shirur et al., 2016; van de Water et al., 2017). Temperate gorgonian corals maintain numerous operational taxonomic units (OTUs) of the same bacterial genus within these dominant phyla (La Rivière et al., 2015). Hosting multiple OTUs of the same genus may allow for “microbial shuffling” of OTUs with similar functions but various stress thresholds, and avoid compromising holobiont fitness by maintaining microbiome function during potentially stressful conditions (van de Water et al., 2017).

In scleractinian corals, exposure to stressors often results in detrimental impacts on their microbial composition as well as to the health of the colonies (Bourne et al., 2008; Ziegler et al., 2017; Gardner et al., 2019). The impacts of stressors on octocoral microbiomes is currently unclear. While temperate gorgonian corals living near waste water pollution had different microbial communities than those in unpolluted areas, how these microbiome differences relate to colony health is still unknown, as visibly healthy colonies, in both the polluted and unpolluted sites, contained the potentially pathogenic *Vibrio shiloi* bacteria (Vezzulli et al., 2013; van de Water et al., 2017, 2018b). In the Great Barrier Reef, exposure to elevated temperatures and decreasing pH did not alter the microbiome of the octocoral *Lobophytum pauciflorum*, nor impacted colony health (Wessels et al., 2017). Within the Caribbean, two studies investigated the potential effects of environmental stressors on gorgonian corals, although neither examined changes in the abundance of different OTUs within the most dominant bacteria. When a natural seawater thermal spike occurred, that led to the bleaching of *Orbicella faveolata*, a scleractinian coral, colonies of the gorgonian *Gorgonia ventalina* did not bleach, although they showed significant shifts in their microbiome (Tracy et al., 2015). Conversely, in the gorgonian corals *Eunicea flexuosa* and *Pseudoplexaura porosa*, no significant differences were found between the bacterial communities in mechanically injured branches and those in healthy uninjured branches within the same colonies, although there were changes in the abundance of some bacteria in and around the damaged tissue (Shirur et al., 2016). These studies identified one abundant bacteria *Endozoicomonas* (order Oceanospirillales) as frequently changing in relation to environmental stressors, although how these fluctuations may relate to the health of octocoral colonies needs to be further investigated.

Gleaning more information about the microbiome of Caribbean gorgonian corals, both at ambient conditions and under potential environmental change, is imperative for our understanding of Caribbean coral reefs. Although scleractinian coral cover within the Caribbean continues to decline (Hughes and Tanner, 2000; Carpenter et al., 2008), gorgonian corals thrive (Tsounis and Edmunds, 2017). Therefore, a goal of our study was to characterize the bacterial composition of Caribbean gorgonian corals at ambient conditions. Our second goal was to determine the effects of elevated seawater temperatures, and/or ultraviolet radiation (UVR), or nutrient enrichment, on the bacterial assemblages of Caribbean gorgonian corals.

## Materials and Methods

Gorgonian octocorals were collected near Puerto Morelos, Mexico. Samples were obtained either from a patch reef in a lagoon (20°50′N, 86°52′W) at 2 m depth or from a back reef at La Bocana (20°52′N, 86°51′W) at 5 m depth.

### Samples for Determination of the Ambient Microbiome in Gorgonian Corals

From the lagoon, six gorgonian species were sampled between July 2012 and May 2015. These gorgonian species were *Pseudoplexaura porosa* (sampled July 2012, 2014, May 2015), *P. flagellosa* (July 2012), *Eunicea tourneforti* (July 2012, 2014, May 2015), *E. flexuosa* (July 2012), *Plexaurella nutans* (July 2014), and *Pterogorgia anceps* (July 2012, 2014). In the back reef, samples were collected from *Pseudoplexaura crucis* and *E. tourneforti* in July and December, 2012. At both sites, eight different colonies, at least 3 m apart, were sampled from each gorgonian species. In each colony, the top 6–10 cm were cut from a randomly chosen branch. Branches were individually placed in bags filled with seawater and transported to the lab where they were flash frozen in liquid nitrogen and stored at −70°C.

### Samples for Determination of the Microbiome in Gorgonian Corals Exposed to Stressors

Short term exposure to UVR and elevated temperature: branches from the gorgonian corals *P. crucis* and *E. tourneforti* were collected from a back reef in July and December 2012 (for detailed methods see McCauley et al., 2018). After an 11-day acclimation period, four branches from each colony were subjected to one of the following four treatments: (1) UVR opaque and ambient temperature, (2) UVR opaque and elevated temperature (+3°C above the ambient seawater temperature), (3) UVR transparent and ambient temperature, and (4) UVR transparent and elevated temperature. The temperature was elevated with submersible heaters to 32 ± 0.5°C in the summer and 29 ± 0.5°C in the winter. UVR exposure was achieved by replacing UVR opaque plates with UVR transparent plates for 4-h increments until complete replacement. The branches were held at the different treatment conditions for 7 days and then immediately flash frozen and stored at −70°C (McCauley et al., 2018).

Short term exposure to sea water enriched with either nitrogen or phosphorous: In May 2015, after an 11-day acclimation period, branches of the gorgonian corals *P. porosa* and *E. tourneforti*, collected from the lagoon, were exposed to either ambient seawater [29 ± 1°C, 1.6–2.0 μM ammonium (NH_4_^+^), 0.8–1 μM phosphate (PO_4_^3–^)] or nitrogen or phosphorous enrichment in flow through aquaria (for detailed methods see McCauley and Goulet, 2019). The enrichment conditions consisted of either 4 ± (0.3) μM phosphate and ambient ammonium, 10 ± (0.6) μM ammonium and ambient phosphate, or 50 ± (1.4) μM ammonium and ambient phosphate. The experiment lasted for 7 days, after which the gorgonian branches from the various treatments and control were flash frozen in liquid nitrogen and stored at −70°C.

### Sample Processing

The frozen branches from all the collections were transported to The University of Mississippi where they were freeze dried and stored at −80°C. Microbiome DNA was extracted either by using a MoBio PowerSoil DNA Isolation Kit (MoBio, Carlsbad, CA, United States) for *E. tourneforti* or, for the other gorgonian species, using the Wizard Genomic DNA Purification Kit (Promega, Madison, WI, United States). Dual-indexed barcoded primers were used to amplify the DNA encoding the V4 regions of the 16S rRNA following established protocols (Kozich et al., 2013; Stone and Jackson, 2016). The amplified 16S rRNA gene fragments were combined and spiked with 5% PhiX (Jackson et al., 2015) before being sequenced on an Illumina MiSeq at The University of Mississippi Medical Center Molecular and Genomics Core facility.

Raw sequence files (FASTQ) were processed using the bioinformatics software mothur version 1.41.1 (Schloss et al., 2009, 2011) following the established pipeline and mothur SOP^1^ referenced on January 2019 (Schloss et al., 2011; Kozich et al., 2013). The Silva database release 132 (Quast et al., 2012) was used to align the sequences. Sequences that contained ambiguous sequences, homopolymers >8 base pairs, were longer than 275 base pairs, or did not align with the V4 region, were discarded. Sequences that did not overlap were filtered out. Remaining sequences were then classified to the RDP version 16 database (Cole et al., 2014). Potential chimeric sequences, and sequences identified as chloroplast, mitochondria, Archaea, Eukarya, or unclassified, were removed from the analysis. The remaining unique bacterial sequences were then grouped into OTUs based on 97% similarity. OTUs that were represented by a single sequence were excluded from diversity analyses, which occurred following subsampling (1,000 iterations) to a standardized number of 1,000 sequence reads per sample.

In mothur, the Bray-Curtis (relative abundance based) index was calculated for each gorgonian coral colony and non-metric multidimensional scaling (NMDS) used to visualize dissimilarity patterns. Spearman’s rank correlation of the relative abundance of OTUs with NMDS axes scores was used to derive the most influential OTUs. Analysis of molecular variance (AMOVA) was used to determine differences between groups of bacterial communities. Repeated measure permutational multivariate analysis of variance (PERMANOVA) was conducted for bacterial data collected over multiple time points using the packages BiodiversityR (Kindt and Coe, 2005) and vegan. Co-occurrence analysis of bacterial OTUs was also conducted on branches collected over multiple timepoints using the co-occurrence_network() function of the microbiomeSeq package (Williams et al., 2014). Strong correlations (ρ > 0.75, *p* < 0.05) between bacterial genera were identified for each time point, with the correlation matrix exported to, and analyzed with, Cytoscape v3.8.0 (Shannon et al., 2003). PERMANOVAs were also used to analyze the seasonal impact of elevated temperature and/or UVR exposure on the bacterial communities of *P. crucis* and *E. tourneforti*, in addition to any interaction when they were combined. Multiple factor analysis of variance (ANOVA) was used to analyze the alpha diversity of branches exposed to elevated temperature and UVR treatments in 2012. OTUs were placed in categories of occurring in 100%, over 50%, less than 50%, and 0% of individual colonies in each gorgonian species. Indicator analysis was conducted on OTUs from gorgonian corals exposed to environmental perturbations. Hierarchical clustering analysis was conducted in R using the hclust() function and visualized with ggplot2 package (Wickham, 2016).

To predict protein pathways present in the bacterial community, 94 OTUs, representing 95.1% of the total sequence count, were uploaded to Piphillin (Iwai et al., 2016). The predicted metagenomes were profiled using the October 2018 KEGG (Kyoto Encyclopedia of Genes and Genomes) database with a 99% cutoff to identify potential protein pathways (Iwai et al., 2016). Significant differences were calculated between the relative abundance of OTUs and KEGG pathways with MANOVA and Tukey’s honest significant difference test, utilizing the OTU cutoff at 0.02% (<100 sequences) of the total sequence count.

## Results

A total of 154 coral colonies from the seven gorgonian species yielded a combined 431,602 bacterial sequences, with an average coverage of 0.94. Two bacterial phyla accounted for almost 95% of the total 16S rRNA gene sequence reads: Proteobacteria (53.6%) and Tenericutes (41.1%). Gammaproteobacteria accounted for 89% of the Proteobacteria sequences, while Mollicutes comprised the entirety of Tenericutes sequences. The remaining 5.3% of the sequence reads predominantly consisted of Bacteroidetes, Cyanobacteria, and unclassified bacteria.

### Ambient Gorgonian Coral Microbiomes

**FIGURE 1:**
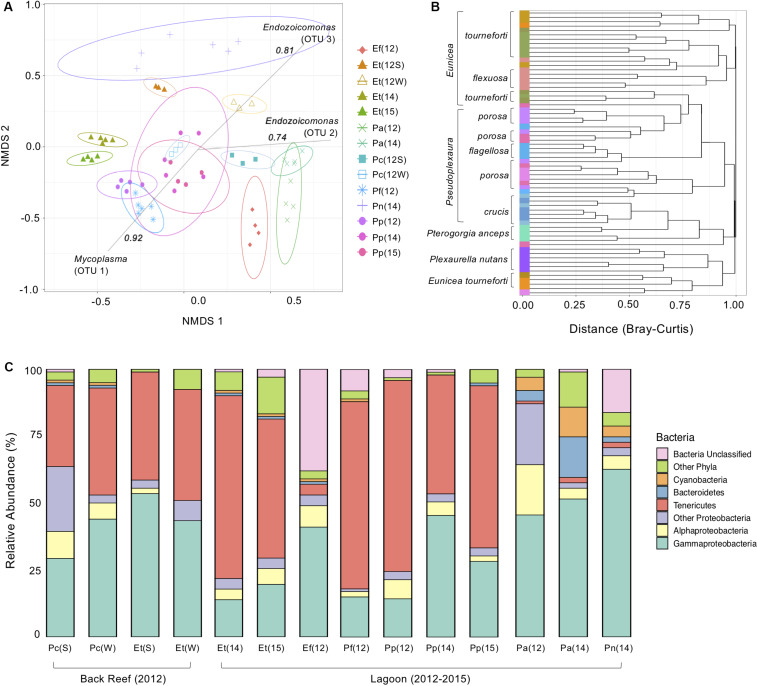
The bacterial microbiome of seven Caribbean gorgonian species in the back reef and lagoon. **(A)** Bacterial beta diversity, shown as non-metric multidimensional scaling ordination (stress = 0.18) based on Bray-Curtis dissimilarity between gorgonians. Ellipses depict 95% confidence intervals and strong (>0.75) Spearman Operational Taxonomic Unit (OTU) correlations included for each significantly correlated OTU. **(B)** Branches are clustered based on Bray-Curtis dissimilarity, and displayed as a hierarchical clustering dendrogram. **(C)** Relative bacterial phyla and class abundance (%) within the gorgonian corals. Each column represents data from 3 to 6 gorgonian coral colonies and from 4,007 to 30,706 sequence reads. The gorgonian species are *Pseudoplexaura crucis* (Pc), *Eunicea tourneforti* (Et), *Eunicea flexuosa* (Ef), *Pseudoplexaura flagellosa* (Pf), *Pseudoplexaura porosa* (Pp), *Pterogorgia anceps* (Pa), and *Plexaurella nutans* (Pn). Numbers in parentheses denote sampling years: 2012 (12), 2014 (14), and 2015 (15), with letters indicating summer (S) or winter (W).

The composition of the bacterial communities varied significantly between the gorgonian coral species sampled in the lagoon (AMOVA, *p* < 0.05 for all, Figure 1A). There was a tight clustering of branches within gorgonian coral species, with phylogenetically similar species clustering closer together, although species that were sampled during different years, didn’t always cluster together (Figure 1B). Bacterial community evenness (Shannon) and diversity (Inverse Simpson) were significantly higher (ANOVA, *p* < 0.05 for both) in *P. anceps* (2012) and in *P. nutans* (2014) when compared to the other gorgonian species collected in the lagoon, which all had similar alpha diversities (Supplementary Table S1). In *E. flexuosa, P. anceps*, and *P. nutans*, Proteobacteria was the most abundant bacterial phylum, with Gammaproteobacteria representing the most common class within it (Figure 1C). Conversely, in *E. tourneforti* and *P. flagellosa*, the majority of bacterial sequences classified as members of the Tenericutes (Figure 1C).

For gorgonian coral species sampled in multiple years, typically the same bacterial phyla were present each year, although there were some differences in their relative abundance. For example, within *P. anceps* the dominant bacterial phyla remained the same from 2012 to 2014 (ANOVA, *p* > 0.05), although significant shifts in their percentage contribution occurred (Figure 1C). Decreases in the proportions of both Alphaproteobacteria and Deltaproteobacteria (Other Proteobacteria, Figure 1C) from 2012 to 2014 coincided with an increase in Gammaproteobacteria (ANOVA, *p* < 0.05 for all). The relative abundance of Bacteroidetes also significantly increased over this time period, as did that of Cyanobacteria and Tenericutes (ANOVA, *p* < 0.05 for all; Figure 1C), although the latter still accounted for just a small proportion of the microbiome in this coral species. Both the Shannon and Inverse Simpson bacterial alpha diversities in *P. anceps* were significantly reduced in 2014 when compared to 2012 (ANOVA, *p* < 0.05; Supplementary Table S1). The microbiome of *P. porosa* also showed temporal differences. The overall bacterial community associated with *P. porosa* in 2014 was significantly different from that in 2012 and 2015 (AMOVA, *p* < 0.001), although there were no significant differences in alpha diversity (ANOVA, *p* < 0.05; Supplementary Table S1). A significantly (ANOVA, *p* < 0.05) lower proportion of Tenericutes in 2014 (and concomitant increase in the proportion of Gammaproteobacteria) compared to 2012 and 2015 likely drove the community-level differences (Figure 1C), as well as a greater proportion of Proteobacteria in 2015 than in 2012 (ANOVA, *p* < 0.05).

While the bacterial composition in *E. tourneforti* from the lagoon did not vary significantly between 2014 and 2015 (AMOVA, *p* < 0.05), they were both significantly different from the bacterial composition in colonies sampled on the back reef (AMOVA, *p* < 0.05), although there were no significant differences in their evenness or diversity (ANOVA, *p* > 0.05, Supplementary Table S1). *E. tourneforti* colonies in the back reef had a significantly higher percentage of Proteobacteria in their microbiome, almost double that present in the lagoon (ANOVA, *p* < 0.01). Within Proteobacteria, the concentration of Gammaproteobacteria in the back reef was nearly three times that of the lagoon, with Oceanospirillales as the dominant order. Enterobacteria accounted for a significantly (ANOVA, *p* < 0.01) larger proportion of the microbiome of *E. tourneforti* sampled from the lagoon (11.2% of the sequences recovered in 2014 and 7.9% of sequences in 2015) compared to those on the back reef (2.7% of sequences).

There were significant seasonal differences in overall bacterial composition of *P. crucis* and *E. tourneforti* sampled from the back reef in 2012 (AMOVA, *p* < 0.05 for both species; Figure 1A). In *P. crucis* colonies, from summer to winter, there was a significant decrease in the percentage of Proteobacteria from 64.9 to 52.9% (ANOVA, *p* < 0.05), although the percentage of Gammaproteobacteria increased from 29.4 to 44.6% (ANOVA, *p* < 0.001), concurrent with an increase in Tenericutes from 30.0 to 40.3% (*p* < 0.05; Figure 1C). In *E. tourneforti* colonies, the opposite shift in dominant bacteria occurred, with a significant increase from summer to winter in the percentage of Proteobacteria from 57.4 to 73.1% (ANOVA, *p* < 0.05) and a decrease in the percentage of Tenericutes from 40.3 to 22.5% ANOVA, (*p* < 0.01; Figure 1C).

**FIGURE 2:**
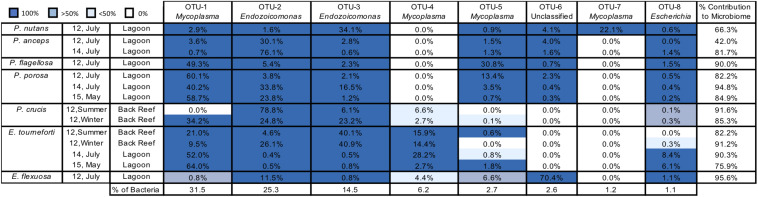
The most abundant bacterial Operational Taxonomic Units (OTUs) in seven Caribbean gorgonian species. Prominent bacterial OTUs (1–8) are listed to the lowest bacterial taxonomic level. OTU presence in gorgonian branches (0–100% of branches sampled) is depicted by different shades of blue. The mean contribution (% of sequences) of each OTU to the microbiome of a gorgonian species is listed in each cell, with the total contribution in the final column. The prevalence (%) of each bacterial OTU in the bacteria obtained from all the gorgonian species combined appears in the bottom row. The gorgonian species are *Plexaurella nutans, Pterogorgia anceps, Pseudoplexaura flagellosa, Pseudoplexaura porosa, Pseudoplexaura crucis, Eunicea tourneforti*, and *Eunicea flexuosa;* Summer = July, Winter = December.

Across the entire dataset, 4,988 OTUs were identified, of which eight contained 361,491 sequences and together accounted for 85.2% of the total reads (Figure 2). OTU 1 was identified as a *Mycoplasma* (Tenericutes, Mycoplasmatales) and OTU 2 and OTU 3 were both identified as belonging to *Endozoicomonas* (Gammaproteobacteria, Oceanospirillales). Combined, these three OTUs accounted for over 70% of the sequences recovered (Figure 2). OTUs 4, 5, and 7 were also classified within *Mycoplasma* and represented 10.1% of all sequences (Figure 2). OTUs identified as an unclassified member of the Firmicutes (OTU 6) and *Escherichia* (OTU 8) represented 2.6% and 1.1% of all bacterial sequences, respectively.

There was a high level of consistency in the OTU composition of the gorgonian coral microbiome. Between all species/locations/time combinations, there was an average of 88.5% (74.8% for the most abundant OTUs) of the microbiome that was composed of OTUs shared between all colonies of each gorgonian species (Figure 2). Every colony sampled, regardless of coral species, contained OTUs identified in the genus *Endozoicomonas* (OTUs 2, 3; Figure 2). With the exception of *P. crucis* sampled from the back reef in summer 2012 and *E. flexuosa* sampled from the lagoon in 2012, every colony sampled also contained OTU 1 (*Mycoplasma*). Every gorgonian colony collected from the lagoon also contained OTU 8, identified as a member of *Escherichia*, but this OTU was not present in all of the colonies sampled from the back reef (Figure 2).

For coral species sampled over different years, the prevalence of bacterial OTUs in individual colonies varied by species. For *P. anceps* and *P. porosa*, the presence of OTUs in each colony was the same from year to year; however, there were changes in some of the relative abundances. For example, OTU 2 (*Endozoicomonas*) accounted for 30.1% of the *P. anceps* microbiome in 2012 but 76.1% in 2014. In *P. porosa* colonies, OTU 2 changed from 3.8% in 2012 to 33.8% in 2014 and then to 23.8% in 2015. *E. tourneforti* also showed year to year changes in the prevalence of some OTUs. For example, every *E. tourneforti* colony sampled in summer 2015 had OTU 5 (*Mycoplasma*), whereas only 50% of the colonies sampled in 2015 contained this OTU (Figure 2).

The co-occurrence of bacterial taxa changed yearly in the three gorgonian species sampled over different summers (Supplementary Table S2). Within branches of *P. anceps* in 2012, the bacterial genus *Pirellulaceae* (Planctomycetes) associated with *Planctomycetes* (Planctomycetes), *Lewinella* (Bacteroidetes), and *Bdellovibrio* (Proteobacteria) in a single module. In 2014, not only was there greater complexity between co-occurring bacteria, within an increased number of modules and paths between nodes, but the majority of these bacteria did not occur in 2012 (Supplementary Table S2). *P. porosa* had a significant reduction in the number and complexity of co-occurring bacteria, reducing from 25 in 2012 to 6 in 2014 and 2015. Further, there was no overlap in the identity of any bacteria across the three timepoints (Supplementary Table S2). In 2014, only members of the phyla Proteobacteria co-occurred within branches of *E. tourneforti*, while in 2015 members of Actinobacteria, Chloroflexi, Tenericutes and Firmicutes also co-occurred (Supplementary Table S2).

*Pseudoplexaura crucis* and *E. tourneforti* were sampled from the back reef in both summer and winter of 2012. With the exception of OTU 1 (*Mycoplasma*) being present in all *P. crucis* colonies sampled during the winter and none in the summer, there were no seasonal changes to the OTUs present in all *P. crucis* colonies (Figure 2). All *E. tourneforti* colonies sampled in the summer harbored OTU 5 (*Mycoplasma*), while less than 50% of colonies sampled during the winter did (Figure 2), but there were no other changes to the OTUs found in every colony across the two seasons.

### Microbial Diversity Under Experimental Conditions

**FIGURE 3:**
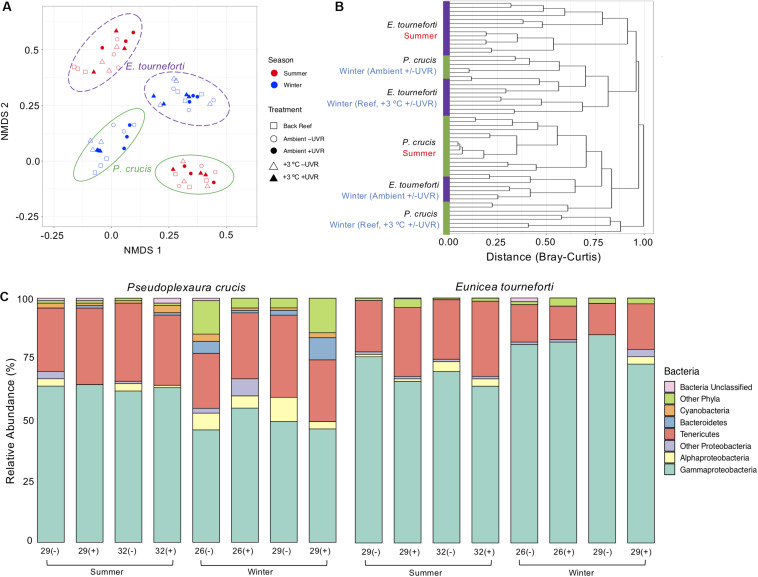
The bacterial microbiome in the two Caribbean gorgonian species *Pseudoplexaura crucis* and *Eunicea tourneforti* sampled from a back reef and after 7 days of experimental exposure to ambient or elevated (+3°C) temperature without (–) or with (+) ultraviolet radiation, in the summer and winter. **(A)** Bacterial beta diversity, shown as non-metric multidimensional scaling ordination (stress = 0.13) based on Bray-Curtis dissimilarity between samples. Ellipses depict the 95% confidence intervals. **(B)** Branches are clustered based on Bray-Curtis dissimilarity, and displayed as a hierarchical clustering dendrogram. **(C)** Relative bacterial phyla and class abundance (%) within the gorgonian coral species. Each column includes from 3,619 to 28,111 sequence reads and represents data from 3 to 4 gorgonian coral colonies.

There were no significant changes to overall bacterial composition in *P. crucis* and *E. tourneforti* following exposure to elevated temperature and/or UVR either during summer or winter. Furthermore, there were no significant interactions between the treatments when combined during either season (PERMANOVA, *R*^2^ < 0.15, *p* > 0.05 for all; Figure 3A). Seasonal effects were more important than the experimental treatments in separating bacterial communities (Figure 3A). All branches collected during the summer clustered tightly together (within species) using the Bray-Curtis dissimilarity index (Figure 3B). During the winter, however, the branches clustered separately, according to the temperature treatment but not UVR (Figure 3B). Bacterial alpha diversity in *P. crucis* and *E. tourneforti* colonies was also not significantly affected by temperature, UVR, or their combination, in either summer or winter (ANOVA, *p* > 0.05 for all; Supplementary Table S1).

**FIGURE 4:**
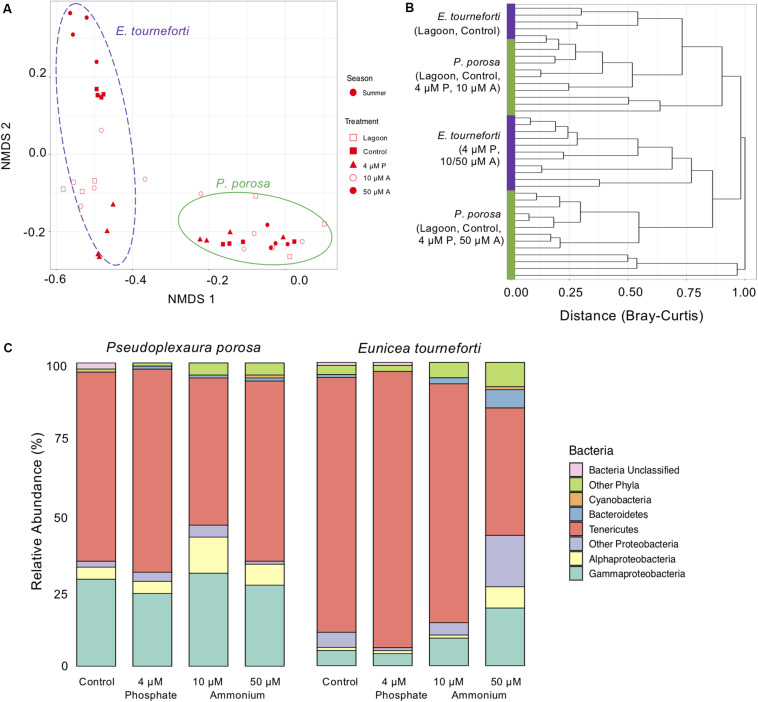
The bacterial microbiome in the two Caribbean gorgonian species *Pseudoplexaura crucis* and *Eunicea tourneforti* sampled from a lagoon and after 7 days of experimental exposure to phosphorous (4 μM) and nitrogen (10 μM and 50 μM) nutrient enrichment. **(A)** Bacterial beta diversity, shown as non-metric multidimensional scaling ordination (stress = 0.09) based on Bray-Curtis dissimilarity between samples. Ellipses depict the 95% confidence intervals. **(B)** Branches are clustered based on Bray-Curtis dissimilarity, and displayed as a hierarchical clustering dendrogram. **(C)** Relative bacterial phyla and class abundance (% of sequences) within the gorgonian corals. Each column includes from 7,525 to 27,886 sequence reads and represents data from 4 to 6 gorgonian coral colonies exposed for 7 days to either no nutrient enrichment (Control), 4 μM phosphate (4 μM P), and 10 μM (10 μM A), or 50 μm (50 μM A) ammonium enrichment.

Colonies did exhibit shifts in the proportion of the represented bacteria (Figure 3C), although these changes varied seasonally. During the winter, in comparison to *P. crucis* branches maintained in the ambient treatment (26°C with UVR), those held at ambient temperature without UVR, and elevated temperature (29°C) with UVR, had decreased proportions of Bacteroidetes in their microbiome (ANOVA, *p* < 0.001) from 4.4 to 0.9% and 0.18% of the microbiome, respectively (Figure 4A). Yet, with the combined exposure of elevated temperature and UVR, there was an increase from the ambient treatment (26°C with UVR), from 4.4 to 8.9% (ANOVA, *p* < 0.05). Branches exposed to ambient temperature without UVR also exhibited increases in the relative abundance of other bacteria (Figure 3C), primarily the Actinobacteria (ANOVA, *p* < 0.02), which increased from 2.0 to 8.0% of the community. Branches at the elevated temperature (29°C) with UVR also showed increases in other bacteria (Figure 3C), primarily the Firmicutes (ANOVA, *p* < 0.03), which increased to 3.2% of the colony microbiome. Changes in the proportions of bacterial phyla within the microbiome of *E. tourneforti* also occurred following some of the treatments in winter (Figure 3C). There was an increase in the relative abundance of Tenericutes within branches of *E. tourneforti* exposed to elevated temperature (29°C) and UVR (ANOVA, *p* < 0.01), with a concomitant decrease in the proportion of Gammaproteobacteria (ANOVA, *p* < 0.01). During the winter, both gorgonian species also showed an increase in the prevalence of OTU 8 (identified as *Escherichia*) when exposed to the combined treatment of elevated temperature and UVR.

During the enrichment experiment, the microbiome of acclimated branches was significantly different from that of branches collected from the lagoon (*p* < 0.05), but similar to that of branches exposed to the control treatment (*p* > 0.05). The nutrient enrichment treatments that led to significant changes to the gorgonian bacterial microbiome were exposure of *P. porosa* to 10 μM ammonium (ANOVA, *p* < 0.05), and exposure of *E. tourneforti* to 50 μM ammonium (ANOVA, *p* < 0.03; Figure 4A). *E. tourneforti* branches sampled from the lagoon and the control treatment clustered together but separate from branches in the other treatments (Figure 4B), while branches of *P. porosa* clustered less tightly, with no clear pattern (Figure 4B). Exposure to 10 μM ammonium increased the proportion of Proteobacteria in the *P. porosa* microbiome from 34.8 to 46.6% (ANOVA, *p* < 0.01) and Firmicutes from less than 0.1 to 3.0% (ANOVA, *p* < 0.001), with a concurrent decrease in the proportion of Tenericutes from 63.1 to 48.8% (ANOVA, *p* < 0.01; Figure 4C). When compared to the ambient treatment, *E. tourneforti* branches enriched with 50 μM ammonium had a significantly greater proportion of Proteobacteria (42.2% compared to 10.9%; ANOVA, *p* < 0.01), Bacteroidetes (6.2% compared to less than 0.1%; ANOVA, *p* < 0.01) and a substantially reduced proportion of Tenericutes (42.2% compared to 84.0%; ANOVA, *p* < 0.01; Figure 4C). The Inverse Simpson index significantly increased with 10 μM (ANOVA, *p* < 0.05) and 50 μM (*p* < 0.01) ammonium enrichments (Supplementary Table S1).

**FIGURE 5:**
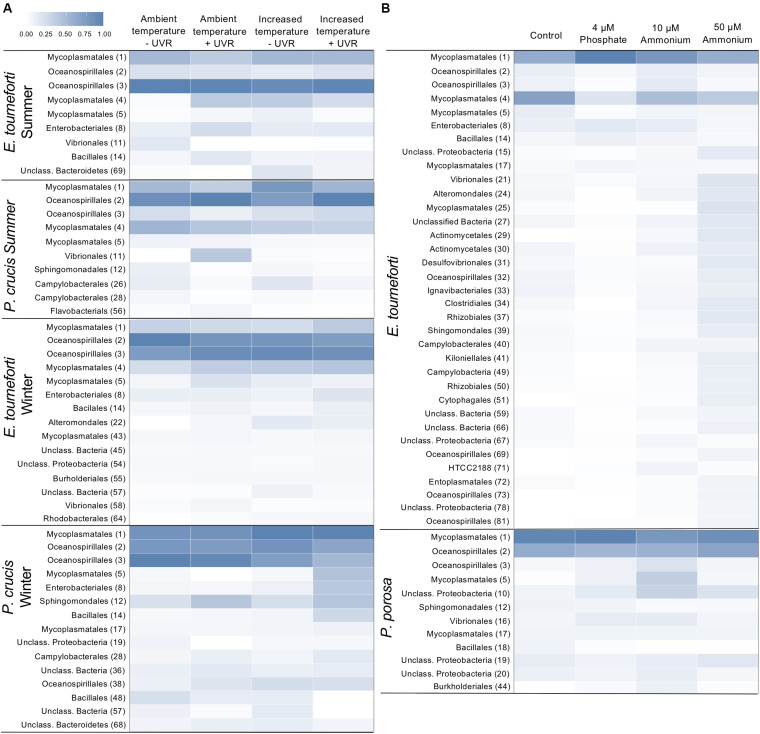
Heat map of the relative abundances of major bacterial Operational Taxonomic Units (OTUs) in the microbiome of Caribbean gorgonian corals. **(A)** *Eunicea tourneforti and Pseudoplexaura crucis* after 7 days exposure to ambient or increased (+3°C) temperatures, without (–) or with (+) ultraviolet radiation (UVR). **(B)** *Eunicea tourneforti* and *Pseudoplexaura porosa* after 7 days exposure to either no nutrient enrichment (Control), 4 μM phosphate, and 10 μM or 50 μM ammonium enrichment. The heat map presents the bacterial orders for OTUs that significantly changed with treatments. Each bacterial order depicted contributed at least 1% of the gorgonian coral’s microbiome assemblage with the OTU number appearing in parentheses. Color strength represents the relative abundance of the OTU, whereby darker blue represents a greater abundance.

There were significant changes to the relative abundance of certain OTUs within the microbiome of both *P. crucis* and *E. tourneforti* when exposed to elevated temperature and/or UVR, both during summer and winter of 2012 (Figure 5A). There was a significant decrease of OTU 2 (a member of Oceanospirillales) and concurrent increase in the abundance of OTU 3 (a member of Oceanospirillales) in branches of *P. crucis* exposed to ambient temperatures without exposure to UVR during the summer, alongside those branches held at increased temperature without UVR (*p* < 0.05; Figure 5A). In *E. tourneforti*, six OTUs that classified as members of Mycoplasmatales, and five that classified as members of the Oceanospirillales underwent significant shifts in abundance with exposure to the experimental treatments (ANOVA, *p* < 0.05 for all; Figure 5A). *E. tourneforti* branches held at ambient temperature (29°C) without exposure to UVR during the summer had a significant increase in the relative abundance of OTU 1 (a member of Mycoplasmatales) while OTU 4 (a member of Mycoplasmatales) decreased, when compared to branches held at ambient temperature with UVR exposure (ANOVA, *p* < 0.05; Figure 5A). A similar response was observed when comparing *E. tourneforti* branches at ambient temperature exposed to UVR with the combined treatment of increased temperature and UVR (ANOVA, *p* < 0.05; Figure 5A). During the winter, when branches of *P. crucis* were exposed to increased temperatures (29°C) without UVR, there was a significant decrease in the abundance of OTU 3 (Oceanospirillales), while two other OTUs within Oceanospirillales (2, 38) significantly increased (*p* < 0.05 for all; Figure 5A). As in the summer, branches of *E. tourneforti* exposed to the ambient temperature (26°C) without UVR had a significant increase in the abundance of OTU 1 (Mycoplasmatales) alongside significant reductions in the abundance of OTU 4 and 5 (Mycoplasmatales) (*p* < 0.05 for all; Figure 5A). Further, when exposed to the combination of increased temperature and UVR, there was an increase in the abundance of OTU 1 (Mycoplasmatales) while OTU 5 (Mycoplasmatales) decreased (*p* < 0.05; Figure 5A).

Under the nutrient enrichments, both *P. porosa* and *E. tourneforti* exhibited shifts in the relative abundance of certain OTUs in their microbiome (Figure 5B). For example, in branches of *P. porosa* exposed to 10 μM ammonium there were decreases in the relative abundance of OTU 1 (Mycoplasmatales) and OTU 2 (Oceanospirillales) but increases in OTU 3 (Oceanospirillales) and 5 (Mycoplasmatales) (*p* < 0.05 for all, Figure 5B). *E. tourneforti* had 11 OTUs, that classified as *Mycoplasma* and Oceanospirillales, increase or decrease in abundance with the various nutrient enrichments (Figure 5B). Branches of *E. tourneforti* that were exposed to the extreme of 50 μM ammonium had significant reductions in the relative abundance of the prominent OTUs 2, and 3 (Oceanospirillales), while there were simultaneous significant increases in the abundance of less dominant OTUs within Oceanospirillales (32, 69, 81, *p* < 0.05; Figure 5B). OTUs 1 and 17 (Mycoplasmatales) significantly increased in relative abundance with exposure to 4 μM phosphate, while OTUs 4, 5, and 25 (Mycoplasmatales) all decreased (*p* < 0.05 for all; Figure 5B). Exposure to 4 μM phosphate also resulted in a significant increase in OTU 8 (Enterobacteriales, identified as *Escherichia*) in *E. tourneforti* (*p* < 0.05) but not in *P. porosa*.

Exposure to 50 μM ammonium led to the exclusive appearance of certain OTUs. In *P. porosa* five exclusive OTUs (0.04–0.25% of sequences recovered) were OTU 30 (*Pseudoalteromonas tunicata*), OTU 35 (*Desulfocella halophila*), OTU 67 (Unclassified Alphaproteobacteria), OTU 96 (Unclassified Rhodobacteraceae), and OTU 103 (*Rubritalea*) (*p* < 0.05 for all). Whereas in *E. tourneforti* there were 25 exclusive OTUs, with the most abundant (0.05–0.16% of sequences recovered) being OTU 26, 49 (*Arcobacter*), OTU 30 (*P. tunicata*), OTU 49 (*Magnetospirillum magnetotacticum*), OTU 51 (*Reichenbachiella*), OTU 65 (*Glaciecola*), OTU 69 (Unclassified Endozoicimonaceae), OTU 75 (Unclassified Kordiimonadaceae), and OTU 96 (Unclassified Rhodobacteraceae) (*p* < 0.05 for all).

### Predicted Protein Pathways in the Gorgonian Coral Microbiome

**FIGURE 6:**
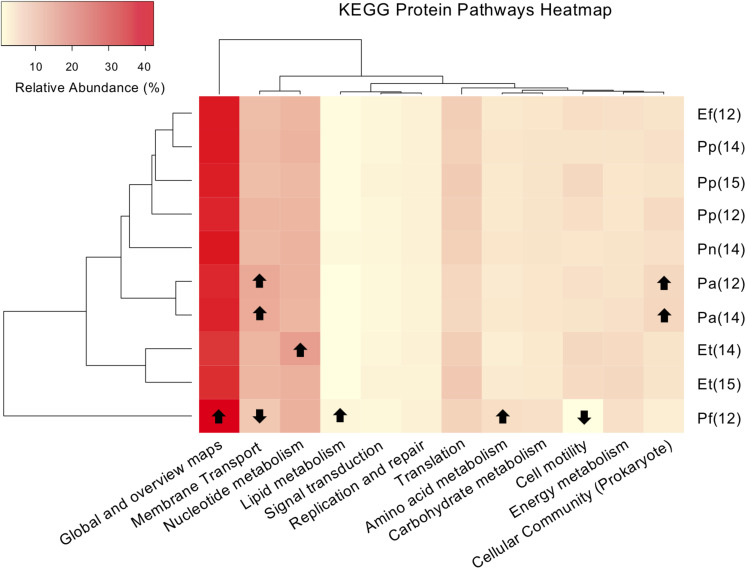
Heat map and dendrogram of the relative abundances (%) of major KEGG (Kyoto Encyclopedia of Genes and Genomes) representative protein pathways present in six Caribbean gorgonian species sampled from a lagoon. The gorgonian species are: *Pseudoplexaura flagellosa* (Pf), *Pseudoplexaura porosa* (Pp), *Eunicea tourneforti* (Et), *Eunicea flexuosa* (Ef), *Pterogorgia anceps* (Pa) and *Plexaurella nutans* (Pn). Numbers in parentheses denote sampling years: 2012 (12), 2014 (14), and 2015 (15). Arrows indicate values significantly higher or lower in relative abundance of the inferred protein pathways present (*p* < 0.05).

295 KEGG protein pathways were predicted from the 16S rRNA data. Bacterial communities from gorgonian corals in the lagoon shared similar ratios of inferred major protein pathways, with an average of 29.4% of genes attributed to global pathways (including metabolic pathways and those related to biosynthesis of secondary metabolites) and nucleotide metabolism and membrane transport pathways each accounting for 10% (Figure 6). Bacteria within *P. flagellosa* had a higher inferred ratio of proteins involved in microbial metabolism, biosynthesis of antibiotics, and metabolism of amino acids and lipids, and lower abundance of genes associated with cell motility and membrane metabolism compared to bacteria present in the other gorgonian species (ANOVA, *p* < 0.05; Figure 6). KEGG analysis of the *P. anceps* microbiome indicated twice the number of prokaryote quorum sensing proteins than any other gorgonian species sampled, in addition to having a greater number of predicted proteins related to photosynthesis and membrane transport, and fewer involved in nitrogen metabolism (ANOVA, *p* < 0.05; Figure 6). Bacteria present within *E. tourneforti* had a higher number of predicted proteins involved in glycolysis, as well as starch and sucrose metabolism (*p* < 0.05). Between 2014 and 2015, there was a significant decrease in the predicted protein abundance involved in nucleotide transport in *E. tourneforti* (*p* < 0.05; Figure 6). There were no significant differences in predicted relative protein abundances in *P. anceps* from 2012 to 2014 (Figure 6). Between samples of *P. porosa* collected from 2012 to 2015, however, there was a significant decrease in the abundance of inferred proteins related to membrane transport, with a significant increase in those related to cell motility in 2015 (*p* < 0.05; Figure 6).

## Discussion

In scleractinian corals, bacteria play critical roles in the health, defense, and functioning of the coral holobiont (Ainsworth et al., 2010, 2015). Compared to our knowledge of over 130 scleractinian bacterial microbiomes, much less is known of the roles of bacteria in octocorals, with reports from only 27 octocoral species. In the Caribbean, the microbiome of five gorgonian species was previously characterized, and our study of seven Caribbean gorgonian species (two of which overlapped with prior studies) concurred with the finding that each gorgonian species hosted a distinct bacterial community (Sunagawa et al., 2010; Correa et al., 2013; Tracy et al., 2015; McCauley et al., 2016; Robertson et al., 2016; Shirur et al., 2016).

At the gorgonian coral genus level, some microbiome similarities existed for the genus *Pseudoplexaura*, with *P. porosa* and *P. flagellosa*, sampled at ambient environmental conditions from the lagoon in the same month and year, hosting very similar bacterial assemblages. On the other hand, the microbiomes of *P. porosa* and *P. flagellosa* were less similar to the microbiome of *P. crucis* collected during the same month, with *P. crucis* having greater proportions of Gammaproteobacteria and unclassified Proteobacteria than either *P. porosa* or *P. flagellosa*. These differences could reflect different collection sites, as *P. crucis* colonies were collected from the back reef while *P. porosa* and *P. flagellosa* colonies were collected from the lagoon. Conversely, even though the two members of the genus *Eunicea*, *E. tourneforti* and *E. flexuosa*, were collected from the same site, they contained very different bacterial assemblages. This incongruity could be due to the two *Eunicea* species differing morphologically. Nevertheless, their branches still clustered together, revealing a greater microbiome similarity to one another than to species from other genera. Phylogeny may also play a role in the observed microbiome similarities and differences, since the *Pseudoplexaura* species sampled are closer phylogenetically to each other than the two *Eunicea* species.

Our findings agree with prior studies on the microbiome of octocorals which found that octocorals often have low bacterial diversity and richness (Bayer et al., 2013a; van de Water et al., 2018a), maintaining a few highly represented bacterial phyla (typically Tenericutes and/or Proteobacteria). At a finer bacterial taxonomic level, members of *Endozoicomonas* and *Mycoplasma* were among the dominant OTUs we detected, and these have previously been found in *P. porosa* and *E. flexuosa* (Shirur et al., 2016) as well as in temperate gorgonian species (reviewed within van de Water et al., 2018a). *Endozoicomonas* is part of the microbiome of many marine invertebrates, often dominating the microbiomes of corals and octocorals (Neave et al., 2016b; van de Water et al., 2017). In scleractinian corals, it can form aggregations adjacent to Symbiodiniaceae (Bayer et al., 2013b). *Endozoicomonas* is likely involved in host associated carbohydrate, protein and sulfur cycling (Neave et al., 2016a; Tandon et al., 2020), with evidence of coevolution with Symbiodiniaceae and corals (La Rivière et al., 2015; Neave et al., 2016b; Wessels et al., 2017).

The specificity of the microbiomes in coral species correlates to characteristics of the holobiont (Grottoli et al., 2018; Pollock et al., 2018). Although the role of *Mycoplasma* in corals remains unclear, it has been previously connected to host feeding, as it was located exclusively within the nematocysts of the coral *Lophelia pertusa* (Neulinger et al., 2009). While *Mycoplasma* may be a parasite in some animals, in temperate gorgonian corals it may be a commensal or mutualistic symbiont, and is often a dominant member of the microbiome of healthy colonies (van de Water et al., 2018a) as our data demonstrate. We found that *P. anceps*, a highly photosynthetic species (Ramsby et al., 2014), had the highest abundance of predicted photosynthetic and membrane transport proteins, but the lowest abundance of predicted nitrogen cycling related proteins, as expected for a highly photosynthetic holobiont. Annually, there were few significant changes to the abundance of inferred proteins present, indicating stability in gorgonian microbiome protein pathways.

Temporally, the dominant OTUs occurred at all sampling times. While there were no changes to the dominant OTUs present in every colony of *P. anceps* and *P. porosa* over the sampling years, there were significant shifts in their proportions. Further, the number of OTUs that co-occurred, alongside their identity and the complexity of these relationships, fluctuated temporally. *P. anceps* showed a proportional increase in *Endozoicomonas* from 2012 to 2014, alongside a 75% increase in Bacteroidetes and a 67% increase Cyanobacteria indicating a potential change in the role of the bacterial assemblage. *Mycoplasma* was dominant in *P. porosa* in 2012 but this dominance shifted to *Endozoicomonas* in 2014, before reverting to *Mycoplasma* in 2015. The decrease in the relative abundance of *Endozoicomonas* in *P. porosa* from 2014 to 2015, occurred concurrently with an increase in the density of the Symbiodiniaceae (*Breviolum* B1i) from 5.16 ± 0.8 × 10^6^/cm^2^ in 2014 to 6.8 ± 0.3 × 10^6^/cm^2^ in 2015 (McCauley and Goulet, 2019).

Seasonal changes in the relative abundances of Tenericutes and Proteobacteria that we found in *P. crucis* and *E. tourneforti* collected from the back reef are similar to seasonal changes reported in the microbiomes of temperate gorgonian corals (van de Water et al., 2018b). This seasonality may reflect changes in gorgonian diets (Cocito et al., 2013), similar to changes in diet influencing the microbial communities of deep sea scleractinian corals (Galand et al., 2020). While the relative abundances of Tenericutes and Proteobacteria changed seasonally, the dominant bacteria generally remained stable. Some shifts in the abundances of the OTUs did occur, a pattern that has been observed in Mediterranean and other Caribbean gorgonian corals (McCauley et al., 2016; van de Water et al., 2017). Temporal changes in the abundances of bacterial communities within gorgonian corals may reveal the holobionts’ ability to respond to changing environmental conditions, potentially via host mediated interactions or through plasticity of the microbial community (reviewed within van de Water et al., 2018a). Given the temporal changes observed in our study, it is possible that these gorgonian coral species and their bacterial consortiums are flexible in their ability to respond to environmental changes, as has been observed in some scleractinian species (Röthig et al., 2016; Ziegler et al., 2017), but not others (Pogoreutz et al., 2018).

Not only were the main OTUs stable temporally, many were spatially stable, shared between gorgonian corals collected from the back reef and from the lagoon. This suggests that gorgonian coral holobionts host stable bacterial assemblages. Importantly, an extremely high percentage of bacteria (60–90%) were shared amongst every individual colony of a particular gorgonian species. Gorgonian corals from the Mediterranean also exhibited high shared bacteria percentages, *Eunicella cavolini* (80.9–90.3%), *E. verrucosa* (44.4–91.5%), *E. singularis* (48.6–87.9%), and *Leptogorgia sarmentosa* (34.4–77.9%) (van de Water et al., 2017). This is in contrast to what is generally found in the tissue of scleractinian corals, whereby scleractinian species do not share many of the same bacteria. For example, 80% of the individuals within the three scleractinian coral species *Mycedium elephantotus*, *Acropora aculeus*, and *Pachyseris speciosa* shared less than 3.0% of their microbiome (Hernandez-Agreda et al., 2018). In addition, in the scleractinian coral *Acropora granulosa* only 0.09% of bacterial OTUs identified were present in 90% of the colonies (Ainsworth et al., 2015).

Furthermore, shared bacterial communities can vary ontogenetically, temporally and spatially as was found in the scleractinian coral *Mussismilia hispida* in which the composition and relative abundance of shared bacterial communities varied from 10.5% of microbial diversity in adult colonies to 84.7% in planula larvae (Leite et al., 2017), seasonally (contributing from 15 to 23% of microbial diversity), and across different reefs with different water qualities (contributing from 23 to 97% of microbial diversity, Leite et al., 2018). One exception to the microbiome commonalities between the gorgonian corals in the back reef vs. the lagoon in our study, was an OTU identified as *Escherichia*, which was present in every colony of every gorgonian species sampled from the lagoon but only in some of the colonies from the back reef. *Escherichia* was also identified in all colonies of *E. flexuosa* and *P. porosa* collected in 2012 in another study (Shirur et al., 2016) from the same lagoon in which we conducted our study. This may be a result of the proximity of the gorgonian corals in the lagoon to sewage released from beachfront hotels in the Mexican Riviera (Baker et al., 2010, 2013). Spirochaetes have been found in gorgonian and scleractinian corals near effluent (van de Water et al., 2016; Wessels et al., 2017) but were not found in our study.

When scleractinian corals are exposed to potential stressors such as elevated temperature, a reduction of beneficial bacteria with a concomitant increase in opportunistic, potentially harmful, bacteria may occur, leading to a detrimental rise in the diversity of the bacterial assemblage (McDevitt-Irwin et al., 2017). These changes may result in a shift from autotrophic to heterotrophic bacterial species which may increase a host coral’s susceptibility to coral diseases, lesions, and tissue necrosis (Meyer et al., 2014; Ainsworth and Gates, 2016; Glasl et al., 2017b; Pootakham et al., 2017; Wessels et al., 2017). On the other hand, when we exposed *P. crucis* and *E. tourneforti* to elevated temperature and/or UVR over 7 days, during either the summer or the winter, there were no significant changes in the dominant bacterial phyla in either, suggesting that their microbiome was resistant or resilient to these thermal and/or UVR treatments. While the overall community assembly did not differ between experimental treatments, changes in specific OTUs did occur, and these changes may relate to both gorgonian species exhibiting a reduction in algal densities during the summer and an increase during the winter (McCauley et al., 2018). Further, the overall stability of the microbiomes is likely related to the fact that although the gorgonian colonies responded to the stressors, observed through non-fatal adjustments in host and algal biochemical parameters in all of the experimental treatments no visible bleaching, tissue loss, or lesions were observed in any of the gorgonian branches (McCauley et al., 2018). Our results concur with the one other octocoral study that examined the effect of temperature stress, for 12 days, on *L. pauciflorum* microbiomes (Wessels et al., 2017). That study found no significant impact on the octocoral microbiome with exposure to reduced pH (Wessels et al., 2017). Furthermore, although in our study in *P. crucis* exposed to elevated temperature and/or UVR, there were increases in the representation of Bacteroidetes, Actinobacteria and Firmicutes, these bacterial phyla are probably involved in nutrient cycling and are likely not detrimental to the host (Cai et al., 2018).

Nutrient enrichment is another stressor that can impact the bacterial consortium of octocorals and scleractinian corals (Vezzulli et al., 2013; Kelly et al., 2014; van de Water et al., 2017; Ziegler et al., 2017), and can increase the severity of diseases in Caribbean gorgonian corals (Bruno et al., 2003). In our short term enrichment, an increase in the proportion of *Endozoicomonas* within *P. porosa* occurred following exposure to 10 μM ammonium, and this coincided with an increase in chlorophyll content and algal density (McCauley and Goulet, 2019). A similar increase in the proportion of *Endozoicomonas* occurred in *E. tourneforti* with exposure to 50 μM ammonium, although this was not matched by an increase in photosynthetic parameters (McCauley and Goulet, 2019). This elevated nutrient enrichment also led to an increase in the proportion of sequences identified as taxa that are typically pathogenic or opportunistic bacteria (e.g., Vibrionales, Alteromonadales, Flavobacteriales). While this indicates that the extreme enrichment conditions resulted in an increase of unhealthy bacteria, they represented less than 3.3% of the gorgonian coral microbiome. Further, none of the gorgonian colonies in any of our experimental treatments showed signs of bleaching or tissue necrosis.

While there were no major shifts in the dominant bacterial genera following the exposure to potential stressors, shifts did occur between different OTUs. van de Water et al. (2018a) hypothesized that such shifts likely lead to gorgonian holobionts restructuring their bacterial community without compromising protein pathway functionality. In our study, we see numerous examples of this, with microbial shuffling occurring in the OTUs identified as *Endozoicomonas* and *Mycoplasma*. This may be an important process by which the gorgonian holobiont could cope and deal with environmental change. Gorgonian corals and their bacterial microbiome respond to changing environments. While some environmental stressors resulted in shifts between OTUs, these shifts may have prevented broader changes to the dominant bacterial phyla present. This ability to respond to the environment, without significantly altering the bacterial community, may be an important reason for why Caribbean gorgonian corals are less susceptible to changing conditions when compared to many scleractinian corals.

## Data Availability Statement

The DNA sequences can be found in the NCBI SRA BioProject, under ID number PRJNA579693.

## Author Contributions

MM, CJ, and TG contributed to the conception and design of the study. MM performed the statistical analysis and wrote the first draft of the manuscript. All authors contributed to manuscript revision, read and approved the submitted version.

## Conflict of Interest

The authors declare that the research was conducted in the absence of any commercial or financial relationships that could be construed as a potential conflict of interest.

## Footnotes

1 https://www.mothur.org/wiki/MiSeq_SOP
