# Supplementary material for: Microbiomes of Caribbean Octocorals Vary Over Time but Are Resistant to Environmental Change
Source: Front Microbiol. 2020 Jun 12;11:1272. doi: 10.3389/fmicb.2020.01272 (PMC7304229; doi:10.3389/fmicb.2020.01272)
Supplement: Supplementary file 1 [file Data_Sheet_1.docx]

|  |  |  |  | Diversity Index | |
| --- | --- | --- | --- | --- | --- |
| Species | Collection Time | Site | Treatment | Inverse Simpson | Shannon |
| *P. nutans* | Summer, 2014 | Lagoon | Field | 5.43 | 2.10 |
| *P. anceps* | Summer, 2012 | Lagoon | Field | 20.98 | 4.12 |
|  | Summer, 2014 | Lagoon | Field | 2.06 | 1.45 |
| *P. flagellosa* | Summer, 2012 | Lagoon | Field | 2.76 | 1.38 |
| *P. porosa* | Summer, 2012 | Lagoon | Field | 2.43 | 1.44 |
|  | Summer, 2014 | Lagoon | Field | 3.38 | 1.57 |
|  | Summer, 2015 | Lagoon | Field | 2.81 | 1.67 |
|  |  |  | Ambient | 2.23 | 1.20 |
|  |  |  | 4 µM P | 2.39 | 1.31 |
|  |  |  | 20 µM A | 3.73 | 1.71 |
|  |  |  | 50 µM A | 2.55 | 1.48 |
| *P. crucis* | Summer, 2012 | Back Reef | Field | 3.64 | 1.74 |
|  |  |  | 29°C (-) | 2.89 | 1.42 |
|  |  |  | 29°C (+) | 2.95 | 1.43 |
|  |  |  | 32°C (-) | 3.11 | 1.54 |
|  |  |  | 32°C (+) | 3.08 | 1.53 |
|  | Winter, 2012 | Back Reef | Field | 4.35 | 2.04 |
|  |  |  | 26°C (-) | 3.76 | 1.50 |
|  |  |  | 26°C (+) | 5.25 | 1.96 |
|  |  |  | 29°C (-) | 4.41 | 1.92 |
|  |  |  | 29°C (+) | 3.76 | 1.69 |
| *E. tourneforti* | Summer, 2012 | Back Reef | Field | 3.41 | 1.79 |
|  |  |  | 29°C (-) | 1.95 | 1.06 |
|  |  |  | 29°C (+) | 2.43 | 1.25 |
|  |  |  | 32°C (-) | 2.60 | 1.45 |
|  |  |  | 32°C (+) | 2.43 | 1.48 |
|  | Winter, 2012 | Back Reef | Field | 3.62 | 1.71 |
|  |  |  | 26°C (-) | 2.63 | 1.19 |
|  |  |  | 26°C (+) | 2.91 | 1.31 |
|  |  |  | 29°C (-) | 2.78 | 1.27 |
|  |  |  | 29°C (+) | 3.46 | 1.58 |
|  | Summer, 2014 | Lagoon | Field | 2.64 | 1.43 |
|  | Summer, 2015 | Lagoon | Field | 3.01 | 1.75 |
|  |  |  | Ambient | 2.87 | 1.61 |
|  |  |  | 4 µM P | 1.28 | 0.55 |
|  |  |  | 20 µM A | 2.88 | 1.63 |
|  |  |  | 50 µM A | 7.51 | 2.39 |
| *E. flexuosa* | Summer, 2012 | Lagoon | Field | 2.47 | 1.41 |

**Supplementary Table 1. Alpha diversity indices Shannon (community evenness) and Inverse Simpson (diversity) for the bacterial microbiome of six Caribbean gorgonian coral species.**

*Plexaurella nutans*, *Pterogorgia anceps*, *Pseudoplexaura flagellosa, Pseudoplexaura porosa*, *Pseudoplexaura crucis, Eunicea tourneforti*, and *Eunicea flexuosa* were sampled during the summer from a Caribbean lagoon (2 m) and were processed immediately. In 2015, *P. porosa* and *E. tourneforti,* sampled from a lagoon, were experimentally exposed to phosphorous (4 µM P) and ammonium (20 µM A and 50 µM A) nutrient enrichment. In 2012, *P. crucis* and *E. tourneforti,* sampled from a back reef (5 m), were experimentally exposed to ambient or elevated (+3ºC) temperature, without (-) or with (+) ultraviolet radiation, both in the summer and winter. Summer samples were collected in July 2012, 2014 and in May 2015. Winter samples were collected in December 2012.

| Gorgonian species | OTU | Taxonomy (Phylum, Class, Genus) | Degree | Centrality |
| --- | --- | --- | --- | --- |
| *Pterogorgia* | 281 | Planctomycetes, Pirellulales, Pirellulaceae unclassified | 7 | 0.95 |
| *anceps* (2012) | 307 | Proteobacteria, Thiohalorhabdales, Thiohalorhabdales unclassified | 2 | 0.00 |
|  | 408 | Proteobacteria, PHOS-HD29 unclassified | 2 | 0.00 |
|  | 615 | Chloroflexi, Ellin6529 unclassified | 1 | 0.00 |
|  | 615 | Proteobacteria, Bdellovibrionales, *Bdellovibrio* | 1 | 0.00 |
|  | 567 | Verrucomicrobia, Verrucomicrobiales, Verrucomicrobiaceae unclassified | 1 | 0.00 |
|  | 355 | Bacteroidetes, [Saprospirales], *Lewinella* | 1 | 0.00 |
|  | 140 | Planctomycetes, Pirellulales, *Planctomycete* | 1 | 0.00 |
|  | 242 | Proteobacteria, BD7-3, BD7-3 unclassified | 1 | 0.00 |
|  | 321 | Proteobacteria, Rhodospirillales, Rhodospirillales unclassified | 1 | 0.00 |
|  | 752 | Chloroflexi, GCA004, GCA004 unclassified | 1 | 0.00 |
|  | 236 | Cyanobacteria, Chroococcales, Chroococcales unclassified | 1 | 0.00 |
|  | 321 | Proteobacteria, Rhodospirillales, Rhodospirillaceae unclassified | 1 | 0.00 |
|  | 1276 | Verrucomicrobia, [Chthoniobacterales], *Candidatus Xiphinematobacter* | 1 | 0.00 |
|  | 224 | Bacteroidetes, Cytophagales, Cytophagales unclassified | 1 | 0.00 |
|  | 1008 | Actinobacteria, Acidimicrobiales, JdFBGBact unclassified | 1 | 0.00 |
|  | 550 | Cyanobacteria, Pseudanabaenales, Pseudanabaenaceae unclassified | 1 | 0.00 |
|  | 934 | Chloroflexi, Anaerolineae unclassified | 1 | 0.00 |
|  | 676 | Planctomycetes, 028H05-P-BN-P5 unclassified | 1 | 0.00 |
|  | 778 | Actinobacteria, Acidimicrobiales, C111 unclassified | 1 | 0.00 |
| *Pterogorgia* | 853 | Proteobacteria, Deltaproteobacteria unclassified | 5 | 0.43 |
| *anceps* (2014) | 72 | Proteobacteria, HTCC2188, HTCC2089 unclassified | 5 | 0.17 |
|  | 73 | Proteobacteria, Gammaproteobacteria, Vibrionales unclassified | 5 | 0.17 |
|  | 90 | Proteobacteria, Alphaproteobacteria unclassified | 5 | 0.17 |
|  | 205 | Fusobacteria, Fusobacteriales, *u114* | 4 | 0.43 |
|  | 114 | Bacteroidetes, Flavobacteriales, *Tenacibaculum* | 4 | 0.43 |
|  | 3 | Proteobacteria, Oceanospirillales, Endozoicimonaceae unclassified | 4 | 1.00 |
|  | 349 | Proteobacteria, PB19, PB19 unclassified | 3 | 0.02 |
|  | 332 | Planctomycetes, Planctomycetales, *Planctomyces* | 3 | 0.02 |
|  | 242 | Proteobacteria, BD7-3, BD7-3 unclassified | 3 | 0.02 |
|  | 120 | Proteobacteria, Rhodobacterales, *Hyphomonas* | 3 | 0.02 |
|  | 104 | Verrucomicrobia, Verrucomicrobiales, *Rubritalea* | 2 | 0.09 |
|  | 102 | Actinobacteria, Actinomycetales, *Dermacoccus* | 2 | 0.09 |
|  | 121 | Proteobacteria, Proteobacteria unclassified | 2 | 0.09 |
|  | 60 | Proteobacteria, Alteromonadales, *Candidatus Endobugula* | 2 | 1.00 |
|  | 471 | Verrucomicrobia, Verrucomicrobiales, *Verrucomicrobium* | 2 | 0.67 |
|  | 21 | Proteobacteria, Vibrionales, Vibrionales_unclassified | 2 | 0.67 |
|  | 676 | Planctomycetes, 028H05-P-BN-P5 unclassified | 1 | 0.00 |
|  | 1178 | Proteobacteria, PHOS-HD29, PHOS-HD29 unclassified | 1 | 0.00 |
|  | 140 | Planctomycetes, Gemmatales, Gemmataceae unclassified | 1 | 0.00 |
|  | 58 | Bacteroidetes, unclassified | 1 | 0.00 |
|  | 22 | Proteobacteria, Alteromonadales, *Alteromonas* | 1 | 0.00 |
|  | 413 | OD1, ZB2 unclassified, ZB2 unclassified | 1 | 0.00 |
|  | 8 | Proteobacteria, Enterobacteriales, Enterobacteriaceae unclassified | 1 | 0.00 |
|  | 89 | Firmicutes, Lactobacillales, *Streptococcus* | 1 | 0.00 |
|  | 57 | Bacteroidetes, Flavobacteriales, *Cloacibacterium* | 1 | 0.00 |
|  | 203 | Proteobacteria, Kiloniellales, Kiloniellales unclassified | 1 | 0.00 |
|  | 45 | Proteobacteria, Pseudomonadales, *Acinetobacter* | 1 | 0.00 |
|  | 37 | Proteobacteria, Rhizobiales, *Methylobacterium* | 1 | 0.00 |
| *Pseudoplexaura* | 21 | Proteobacteria, Vibrionales, Vibrionales unclassified | 5 | 0.50 |
| *porosa* (2012) | 48 | Proteobacteria, Rhizobiales, *Magnetospirillum* | 4 | 0.17 |
|  | 334 | Bacteroidetes, Sphingobacteriales, *Pedobacter* | 3 | 0.03 |
|  | 754 | [Thermi], Deinococcales, *Deinococcus* | 3 | 0.03 |
|  | 89 | Firmicutes, Lactobacillales, *Streptococcus* | 2 | 0.03 |
|  | 77 | Bacteroidetes, Flavobacteriales, Flavobacteriaceae unclassified | 2 | 0.03 |
|  | 47 | Firmicutes, Bacillales, *Staphylococcus* | 2 | 0.00 |
|  | 101 | Proteobacteria, Enterobacteriales, Enterobacteriaceae unclassified | 2 | 0.00 |
|  | 96 | Enterobacteriaceae, Rhodobacterales, Rhodobacteraceae unclassified | 2 | 0.00 |
|  | 1873 | Cyanobacteria, MLE1-12, MLE1-12 unclassified | 1 | 0.00 |
|  | 2810 | Proteobacteria, Ellin329, Ellin329 unclassified | 1 | 0.00 |
|  | 407 | Proteobacteria, Salinisphaerales, *Salinisphaera* | 1 | 0.00 |
|  | 2372 | Proteobacteria, Methylophilales, *Methylobacillus* | 1 | 0.00 |
|  | 393 | Proteobacteria, Caulobacterales, *Brevundimonas* | 1 | 0.00 |
|  | 876 | Verrucomicrobia, [Pedosphaerales], [Pedosphaerales] unclassified | 1 | 0.00 |
|  | 53 | Proteobacteria, Vibrionales, Vibrionales unclassified | 1 | 0.00 |
|  | 173 | Actinobacteria, Actinomycetales, ACK-M1 unclassified | 1 | 0.00 |
|  | 575 | Proteobacteria, Burkholderiales, *Limnobacter* | 1 | 0.00 |
|  | 2 | Proteobacteria, Oceanospirillales, Endozoicimonaceae unclassified | 1 | 0.00 |
|  | 130 | Endozoicimonaceae, Pseudomonadales, *Pseudomonas* | 1 | 0.00 |
|  | 235 | Proteobacteria, Sphingomonadales, Erythrobacteraceae unclassified | 1 | 0.00 |
|  | 459 | Proteobacteria, Betaproteobacteria unclassified | 1 | 0.00 |
|  | 725 | Bacteroidetes, Bacteroidales, *Bacteroides* | 1 | 0.00 |
|  | 111 | Proteobacteria, Legionellales, Francisellaceae unclassified | 1 | 0.00 |
|  | 140 | Planctomycetes, Gemmatales, Gemmataceae unclassified | 1 | 0.00 |
| *Pseudoplexaura* | 222 | Planctomycetes, Pirellulales, *Planctomycete* | 1 | 0.00 |
| *porosa* (2014) | 798 | Proteobacteria, Rhodobacterales, *Paracoccus* | 1 | 0.00 |
|  | 193 | Bacteroidetes, Cytophagales, Cytophagaceae unclassified | 1 | 0.00 |
|  | 345 | Cyanobacteria, Synechococcales, *Synechococcus* | 1 | 0.00 |
|  | 88 | Actinobacteria, Actinomycetales, *Corynebacterium* | 1 | 0.00 |
|  | 173 | Actinobacteria, Actinomycetales, ACK-M1 unclassified | 1 | 0.00 |
| *Pseudoplexaura* | 104 | Verrucomicrobia, Verrucomicrobiales, Verrucomicrobiaceae unclassified | 1 | 0.00 |
| *porosa* (2015) | 568 | Proteobacteria, Rhodobacterales, *Loktanella* | 1 | 0.00 |
|  | 33 | Proteobacteria, Oceanospirillales, *Oleibacter* | 1 | 0.00 |
|  | 230 | Firmicutes, Bacillales, *Bacillus* | 1 | 0.00 |
|  | 91 | Proteobacteria, Desulfovibrionales, *Desulfovibrio* | 1 | 0.00 |
|  | 92 | Proteobacteria, Burkholderiales, Oxalobacteraceae unclassified | 1 | 0.00 |
| *Eunicea* | 164 | Proteobacteria, Rhodobacterales, Rhodobacteraceae unclassified | 1 | 0.00 |
| *tourneforti* (2014) | 520 | Proteobacteria, Alteromonadales, *Shewanella* | 1 | 0.00 |
|  | 225 | Proteobacteria, Xanthomonadales, *Stenotrophomonas* | 1 | 0.00 |
|  | 142 | Proteobacteria, Alteromonadales, OM60 unclassified | 1 | 0.00 |
|  | 174 | Proteobacteria, Pseudomonadales, *Enhydrobacter* | 1 | 0.00 |
|  | 55 | Proteobacteria, Burkholderiales, Comamonadaceae unclassified | 1 | 0.00 |
|  | 37 | Proteobacteria, Rhizobiales, Rhizobiales unclassified | 1 | 0.00 |
|  | 192 | Proteobacteria, Rhizobiales, Hyphomicrobiaceae unclassified | 1 | 0.00 |
| *Eunicea* | 217 | Actinobacteria, Actinomycetales, Microbacteriaceae unclassified | 1 | 0.00 |
| *tourneforti* (2015) | 752 | Chloroflexi, GCA004 unclassified | 1 | 0.00 |
|  | 4 | Tenericutes, Mollicutes unclassified, Mollicutes unclassified | 1 | 0.00 |
|  | 46 | Proteobacteria, Rhodobacterales, Rhodobacteraceae unclassified | 1 | 0.00 |
|  | 8 | Proteobacteria, Enterobacteriales, Enterobacteriaceae unclassified | 1 | 0.00 |
|  | 14 | Firmicutes, Bacillales, Bacillaceae unclassified | 1 | 0.00 |

**Supplemental Table 2. Co-occurring bacterial Operational Taxonomic Units (OTUs) and their taxonomic identification in three repeatedly sampled Caribbean gorgonian coral species.** Gorgonian colonies (n = 4-7 per species) were collected in July 2012, July 2014, and May 2015. Bacterial OTUs are ordered by Degree (number of undirected edges) and by Betweenness centrality.
